# Prefrontal glutamate levels predict altered amygdala–prefrontal connectivity in traumatized youths

**DOI:** 10.1017/S0033291718002519

**Published:** 2018-09-18

**Authors:** Olga Therese Ousdal, Anne Marita Milde, Alexander R. Craven, Lars Ersland, Tor Endestad, Annika Melinder, Quentin J. Huys, Kenneth Hugdahl

**Affiliations:** 1 Department of Radiology, Haukeland University Hospital, Bergen, Norway; 2 Wellcome Trust Centre for Neuroimaging, University College London, London, UK; 3 Department of Biological and Medical Psychology, University of Bergen, Bergen, Norway; 4 Regional Centre for Child and Youth Mental Health and Child Welfare, UNI Research Health, Bergen, Norway; 5 NORMENT, Centre of Excellence, University of Oslo, Oslo, Norway; 6 Department of Clinical Engineering, Haukeland University Hospital, Bergen, Norway; 7 Institute of Psychology, University of Oslo, Oslo, Norway; 8 Translational Neuromodeling Unit, Institute of Biomedical Engineering, University of Zürich and Swiss Federal Institute of Technology (ETH) Zürich, Zurich, Switzerland; 9 Department of Psychiatry, Centre for Addiction Disorders, Psychotherapy and Psychosomatics, Hospital of Psychiatry, University of Zürich, Zurich, Switzerland; 10 Division of Psychiatry, Haukeland University Hospital, Bergen, Norway; 11 KG Jebsen Centre for Neuropsychiatric Disorders, University of Bergen, Bergen, Norway

**Keywords:** Amygdala, functional MRI, glutamate, prefrontal cortex, PTSD, stress

## Abstract

**Background:**

Neurobiological models of stress and stress-related mental illness, including post-traumatic stress disorder, converge on the amygdala and the prefrontal cortex (PFC). While a surge of research has reported altered structural and functional connectivity between amygdala and the medial PFC following severe stress, few have addressed the underlying neurochemistry.

**Methods:**

We combined resting-state functional magnetic resonance imaging measures of amygdala connectivity with *in vivo* MR-spectroscopy (^1^H-MRS) measurements of glutamate in 26 survivors from the 2011 Norwegian terror attack and 34 control subjects.

**Results:**

Traumatized youths showed altered amygdala–anterior midcingulate cortex (aMCC) and amygdala–ventromedial prefrontal cortex (vmPFC) connectivity. Moreover, the trauma survivors exhibited reduced levels of glutamate in the vmPFC which fits with the previous findings of reduced levels of Glx (glutamate + glutamine) in the aMCC (Ousdal *et al*., 2017) and together suggest long-term impact of a traumatic experience on glutamatergic pathways. Importantly, local glutamatergic metabolite levels predicted the individual amygdala–aMCC and amygdala–vmPFC functional connectivity, and also mediated the observed group difference in amygdala–aMCC connectivity.

**Conclusions:**

Our findings suggest that traumatic stress may influence amygdala–prefrontal neuronal connectivity through an effect on prefrontal glutamate and its compounds. Understanding the neurochemical underpinning of altered amygdala connectivity after trauma may ultimately lead to the discovery of new pharmacological agents which can prevent or treat stress-related mental illness.

## Introduction

Stress influences the development and expression of a range of mental disorders, and is a defining feature of trauma- and stressor-related disorders, including post-traumatic stress disorder (PTSD) (Hariri and Holmes, 2015). Disorders associated with stress are among the most common and deliberating mental disorders worldwide, and carries an enormous economic burden on the society (Whiteford *et al*., 2013). Understanding how stress impacts normal brain function and alters the risk for mental illness is therefore of central importance. Despite the increasing knowledge concerning the neuronal circuits altered by traumatic stress exposure (Pitman *et al*., 2012; Tottenham and Galvan, 2016; Herringa, 2017), little is known regarding the underlying neurochemical mechanisms, of which the neuronal circuit changes are a likely consequence.

Research across species indicates that chronic or extreme stress has long-term impact on neuronal networks implicated in emotion-generation and regulation (Arnsten, 2015; McEwen *et al*., 2016; Tottenham and Galvan, 2016; Herringa, 2017). While the connections between amygdala and the dorsal anterior cingulate (dACC)/anterior midcingulate cortex (aMCC) are crucially involved in the processing and generation of emotions, research on emotional regulation converges on interactions between the amygdala and the ventromedial prefrontal cortex (vmPFC) (Etkin *et al*., 2011). Long-term changes in these networks have been observed even after a single episode of extreme stress (van Wingen *et al*., 2012), as well as in patients with stress-related disorders (Pitman *et al*., 2012). This suggests that these connections are central in mediating a transition from stress exposure to stress-related psychopathology. However, the altered connectivity may primarily reflect abnormalities in glutamatergic neurotransmission within long-range amygdala–prefrontal connections, as these bidirectional connections are primarily glutamatergic (Sah *et al*., 2003), and as glutamate levels in prefrontal cortex (PFC) are associated with cortical–subcortical connectivity in humans (Duncan *et al*., 2013).

A rich animal literature suggests that acute and chronic stress, especially stress-induced release of glucocorticoids, induces changes in glutamatergic neurotransmission and levels in the PFC (Popoli *et al*., 2012). Although previous studies from our group (Ousdal *et al*., 2017) and others (Meyerhoff *et al*., 2014; Pennington *et al*., 2014; Yang *et al*., 2015) lend support to an association between severe stress exposure and altered excitatory neurotransmitter levels, the link between these perturbations and the observed changes in prefrontal activation and connectivity remains largely unresolved. Resting-state functional magnetic resonance imaging (rsfMRI) enables approximation of neuronal connectivity by assessing the temporal covariation of low-frequency fluctuations of the blood oxygen level-dependent signal (Biswal *et al*., 1995). By combining rsfMRI with *in vivo* measurements of prefrontal glutamate (Glu) levels using MR spectroscopy (^1^H-MRS), it would be possible to test if the frequently observed perturbations of amygdala–prefrontal connectivity in traumatized subjects are mediated by glutamatergic mechanisms.

Here, we studied survivors from the 2011 Norwegian terrorist attack, a unique group of mainly young adults who all experienced a severe psychological trauma during their late adolescence (Dyb *et al*., 2014; Melinder *et al*., 2015). Based on previous studies of trauma-exposed individuals, we hypothesized that the trauma survivors would exhibit long-term changes in amygdala–prefrontal functional connectivity (van Wingen *et al*., 2012; Thomason *et al*., 2015). Moreover, we tested a contemporary mechanistic model of how changes in amygdala connectivity may occur (Graybeal *et al*., 2012) by linking functional connectivity to regional levels of Glu and the combined levels of Glu and its metabolic product, glutamine (=Glx). Furthering our understanding of the neurochemical underpinning of large-scale brain network connectivity changes in trauma-exposed individuals may ultimately reveal underlying synaptic mechanisms which can be targeted by novel treatments for trauma- and stress-related mental illnesses.

## Methods and Materials

### Subjects

The study was approved by the Norwegian Regional Committees for Medical and Health Research Ethics South East (#2012/1464) and complied with the Helsinki Declaration of 1975, as revised in 2008. Twenty-six survivors from the terror attack at Utøya and 34 healthy control subjects between 16 and 25 years were included in the study after giving written informed consent. All data were collected between 21 and 33 months after the terror attack. This study is part of a larger project assessing the effects of traumatic stress during late adolescence on cognition, behaviour and biological measures.

The trauma survivors were recruited through written invitation sent out from the Resource Centre for Violence, Traumatic Stress and Suicide Prevention, Western Norway (64% response rate). The control sample was an age-, sex- and education-matched group, who were not involved in the trauma, and were not otherwise related to any of the survivors. In order to obtain information concerning participants’ mental health, the Mini International Neuropsychiatric Interview (MINI, 6.0.0; Sheehan *et al*., 2009) was administered. The MINI is a short structured interview that explores psychiatric diagnosis according to the Diagnostic and Statistical Manual of Mental Disorders, Fourth Edition (DSM-IV) and International Classification of Diseases, 10th Edition (ICD-10), and is known for its applicability and sufficient validity and reliability (Sheehan *et al*., 1998).

General exclusion criteria were a history of severe somatic illness, head trauma, ongoing substance abuse and MRI incompatibility. Additional exclusion criteria for the control subjects included history of psychiatric disorders or previous psychological traumas as detected by the MINI. Following initial assessments, five subjects were excluded from the control group due to a history of psychiatric disorder or recent drug use. Furthermore, one subject was excluded from the trauma group based on incidental observation of brain pathology. Finally, two trauma survivors and two controls were excluded due to excessive movement during the scan. The final sample sizes were thus 23 trauma survivors and 27 controls. All subjects received an honorarium of 500 NOK. See Table 1 for further subject characteristics.

**Table 1.** Characteristics of the subjects

Characteristic	Controls (*N* = 27)		Trauma survivors (*N* = 23)		
	*N*	%	*N*	%	*p*^a^
Female	16	59.26	16	69.57	0.56
Age	20.19	s.d.: 2.12	19.57	s.d.: 1.38	0.22
Years of education	13.84	s.d.: 1.69	13.45	s.d.: 1.05	0.34
PTSD	0	0	5	21.74	0.02
Major depressive episode	0	0	3	13.04	0.09
Panic disorder	0	0	8	34.78	0.001
Generalized anxiety disorder	0	0	2	8.70	0.21
Obsessive compulsive disorder	0	0	1	0.04	0.46

a The χ^2^ test was used for sex and psychopathology comparisons across the two groups; two-sample *t* test was used for age and years of education comparisons.

### MRI and analysis

#### fMRI data acquisition and analysis

All images were acquired with a GE Signa HDx, 3 T MR scanner. A detailed description of fMRI data acquisition is provided in the online Supplementary Methods. Data pre-processing was conducted using the SPM12 software package (http://www.fil.ion.ucl.ac.uk/spm). All volumes were realigned to the first volume (Friston *et al*., 1995) and unwarped for correction of head movements and related image distortions. Resting-state fMRI data can be severely affected by head movements, even if standard *post*-*hoc* motion corrections methods are applied. To reduce the influence of motion, we subsequently applied the algorithms implemented in the ArtRepair toolbox to detect and repair bad volumes (http://cibsr.stanford.edu/tools/human-brain-project/artrepair-software.html). We used the software default measures, which included (1) >1.5% variance in global signal from scan to scan and (2) >0.5 mm/TR frame-wise displacement. Volumes that exceeded one of these cut-offs were replaced via interpolation. Subjects with more than 20% bad volumes in total were excluded (Redcay *et al*., 2012), which in our sample included two subjects from the control group and two subjects from the trauma survivors. The number of outlier volumes did not differ significantly between the groups (*t*_48_ = 1.76, *p* = 0.09). After artefact correction, all images were spatially normalized to a standard EPI template based on the Montreal Neurological Institute (MNI) reference brain (Evans *et al*., 1992), and resampled to a voxel size of 3 × 3 × 3 mm. The images were smoothed using an 8 mm full width–half maximum Gaussian isotropic kernel.

**Fig. 1.**
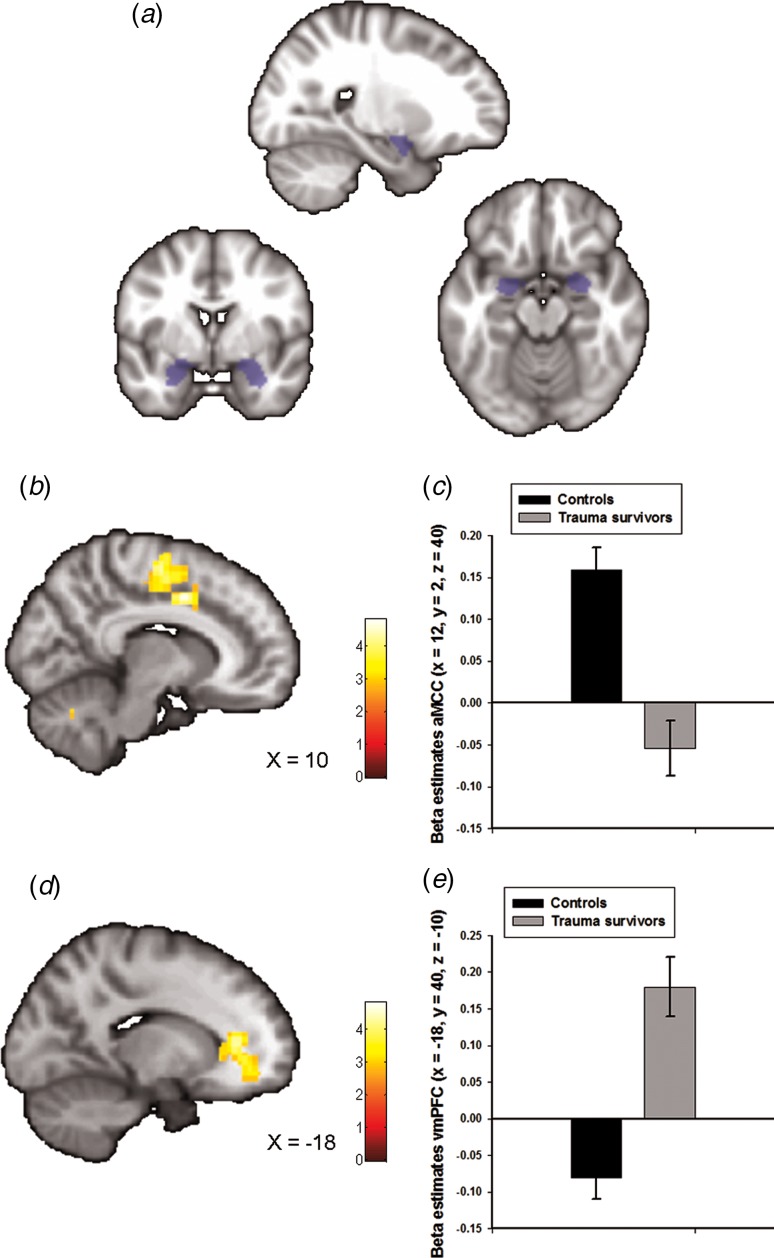
Group differences in amygdala–prefrontal functional connectivity. (*a*) The anatomically defined bilateral amygdala seed region used for functional connectivity analyses. (*b*) Statistical parametric maps (SPM) demonstrating the cluster in anterior midcingulate cortex (aMCC) which showed reduced positive functional connectivity with the amygdala in the trauma survivors. The image is whole-brain FWE cluster corrected. (*c*) Average connectivity strength extracted from the peak voxel in aMCC separated by group. (*d*) SPM demonstrating the cluster in ventromedial prefrontal cortex (vmPFC) which showed reduced negative functional connectivity with the amygdala in the trauma survivors. The image is whole-brain FWE cluster corrected. (*e*) Average connectivity strength extracted from the peak voxel in vmPFC separated by group.

The goal of our analysis was to assess amygdala–prefrontal resting-state connectivity due to the importance of these neurocircuits in stress-related psychiatric disorders (Etkin and Wager, 2007; Pitman *et al*., 2012), and previous reports of altered amygdala connectivity following early life traumas (Thomason *et al*., 2015). To define the amygdala region-of-interest (ROI), we used bilateral masks obtained from the Wakefield Forest University (WFU) atlas (Maldjian *et al*., 2003) (see Fig. 1*a*). Given that we had no *a priori* hypothesis on lateralization, we averaged the signals from the right and the left amygdala masks. Functional connectivity of the amygdala was determined by seed-voxel correlation mapping implemented in the CONN-fMRI toolbox 16b for SPM (https://www.nitrc.org/projects/conn). This method calculated the temporal correlation between brain activation from our seed region and all other brain areas using a General Linear Model (GLM) approach. Following the CompCor strategy as implemented in CONN, nuisance covariates including CSF, white-matter signals and the individual realignment parameters were modelled and regressed out from the analysis. In addition, the data were band-pass filtered (0.008–0.09 Hz).

Subject-specific contrast images reflecting Fisher-transformed correlation values were entered in a second-level random-effects analysis in SPM. Amygdala functional connectivity across the whole sample was investigated in a one-sample *t* test. Then, we compared amygdala functional connectivity for the two groups in a two-sample *t* test. To ensure that the group effect was not driven by the two most common axis 1 disorders in the trauma survivors (see Table 1), we also performed a multiple regression analysis which in addition to a variable coding for group, also contained variables coding for comorbid PTSD and panic disorder. Secondly, we re-analysed the data without the use of motion ‘scrubbing’ to ensure that the reduction in number of datapoints resulting from this procedure did not influence the results. The analysis pipeline was the same as mentioned above, with the exception of any data interpolation as implemented in the ArtRepair software. Finally, to establish the anatomical specificity of our findings, we also investigated group differences in posterior cingulate cortex connectivity. The posterior cingulate cortex ROIs were defined using bilateral Brodmann area 31 (BA31) masks obtained from the WFU atlas.

We tested for statistical significance using an initial voxel-wise threshold of *p* < 0.005 uncorrected, combined with a whole-brain family-wise error (FWE)-corrected significance of *p* < 0.05 at the cluster level. In addition, as we had *a priori* hypotheses regarding amygdala–dACC/aMCC and amygdala–vmPFC connectivity, small volume correction (svc) based on anatomically defined aMCC and vmPFC ROIs and peak-level FWE-corrected *p* values were used. The anatomically defined ROIs were created using the SPM Wake Forest University (WFU) Pickatlas toolbox (Maldjian *et al*., 2003), and consisted of a BA24 mask for the aMCC and a combined BA10/BA32 mask for the vmPFC. Owing to the use of two separate *a priori* masks, a Bonferroni correction of our *α*-threshold was performed for the number of masks (*p*_FWE_ < 0.025).

### Magnetic resonance spectroscopy (^1^H-MRS) acquisition and analysis

The amygdala is closely connected to the vmPFC and the dACC/aMCC (Etkin *et al*., 2011), thus we chose these two anatomical regions *a priori* for measurements of glutamate levels and related compounds. We obtained ^1^H-spectra from the vmPFC and the aMCC using a single voxel point resolved spectroscopy (PRESS) sequence. Glx data from the aMCC and the vmPFC voxels have been published previously (Ousdal *et al*., 2017). A detailed description of ^1^H-MRS data acquisition and analyses are provided in the online Supplementary Methods. Resting-state Glu and Glx levels relative to creatine (Cr) were used from the LCModel output. There was an association between Glu/Cr and white as well as grey matter in the vmPFC MRS voxel (see online Supplementary Methods), thus we controlled for voxel white and grey matter in all analyses of the vmPFC Glu/Cr data. We did not obtain aMCC ^1^H-MRS spectra from three of the controls. Furthermore, vmPFC ^1^H-spectra data from one trauma survivor and two controls had to be excluded due to poor data quality. Thus, the final sample size for the aMCC ^1^H-MRS data included 24 controls and 23 trauma survivors, and for the vmPFC ^1^H-MRS data, 25 controls and 22 trauma survivors.

### Statistical analysis of the combined rsfMRI/^1^H-MRS data

To test if the group difference in aMCC Glx levels mediated the group difference in amygdala–aMCC connectivity, we used hierarchical linear regression as outlined in Baron and Kenny (Baron and Kenny, 1986). To estimate the indirect effects in the mediation model, we used the INDIRECT software as implemented in SPSS (Preacher and Hayes, 2008). To avoid non-independence effects, individual amygdala–aMCC peak connectivity estimates were extracted from an anatomically defined aMCC region (BA 24 from the WFU pickatlas (Maldjian *et al*., 2003)). An equivalent mediation analysis was used to test if the group difference in vmPFC Glu levels mediated group differences in amygdala–vmPFC functional connectivity. The analyses controlled for individual differences in vmPFC grey and white matter. The amygdala–vmPFC peak connectivity estimates were extracted from an anatomically defined vmPFC region (BA 32 from the WFU pickatlas (Maldjian *et al*., 2003)). Indirect effects were considered significant if the 95% confidence interval (CI) did not overlap zero (Preacher and Hayes, 2008).

## Results

Trauma survivors and the controls were well-matched on age, gender and years of education (see Table 1). In total, 12 survivors reported symptoms that met criteria for a psychiatric disorder as assessed in the MINI interview (see Table 1). Among these 12, four subjects had two or more ongoing disorders. None of the trauma survivors reported a prior diagnosis of a mental disorder preceding the terrorist attack. One trauma survivor occasionally used a low-dose benzodiazepine for insomnia; otherwise none of the survivors were prescribed any medications.

### rsfMRI connectivity

Amygdala functional connectivity maps across groups are presented in online Supplementary Fig. S1 with corresponding statistics in online Supplementary Table S1. We observed whole-brain significant group differences in amygdala–aMCC connectivity (peak voxel: *x* = 12, *y* = 2, *z* = 40, *t* = 5.00, *p*_FWE_ < 0.001, Fig. 1*b* and online Supplementary Fig. S2a), amygdala–vmPFC (peak voxel: *x* = −18, *y* = 40, z = −10, *t* = 4.82, *p*_FWE_ < 0.001, Fig. 1*d* and online Supplementary Fig. S2*b*), amygdala–dorsolateral PFC (peak voxel: *x* = 40, *y* = −10, *z* = 44, *t* = 4.33, *p*_FWE_ = 0.01) and amygdala–cerebellum (peak voxel: *x* = 0, *y* = −80, *z* = −38, *t* = 4.60, *p*_FWE_ = 0.02) connectivity. The location of the aMCC cluster accords with a recent meta-analysis which showed that hyperactivity in the aMCC is among the most consistent finding in traumatized subjects which develop PTSD (Hayes *et al*., 2012). Extraction of average connectivity signal strength within the aMCC and the vmPFC clusters revealed that while amygdala–aMCC functional connectivity was positive in the control group, there was a loss of positive connectivity in the trauma survivors (Fig. 1*c*). Moreover, the trauma survivors had less negative connectivity between the amygdala and the vmPFC (Fig. 1*e*), compared with the healthy controls. To control for group differences in the most common axis 1 disorders, we also performed a multiple regression analysis assessing the association between group (trauma survivors *v.* controls) and amygdala connectivity while controlling for comorbid PTSD and panic disorder. The group differences were robust to adjustments for comorbidity (see online Supplementary Table S2). Except for the group difference in amygdala–dorsolateral PFC connectivity, we also replicated the findings in un-scrubbed data (see online Supplementary Table S3). Thus, the main findings were not dependent on any adjustments made by the ArtRepair software. Finally, a comparison of BA31 functional connectivity between the groups revealed no whole-brain or small-volume significant clusters.

If the altered amygdala functional connectivity in the trauma group is driven by their traumatic experiences *per se*, we might expect the individual amygdala connectivity to be related to time elapsed since the traumatic event. To test this, we calculated for each subject the number of days between times of testing and the traumatic event. Next, these scores were regressed onto the individual functional connectivity maps in SPM. The analysis revealed no whole-brain or small-volume significant clusters.

### ^1^H-MRS

**Fig. 2.**
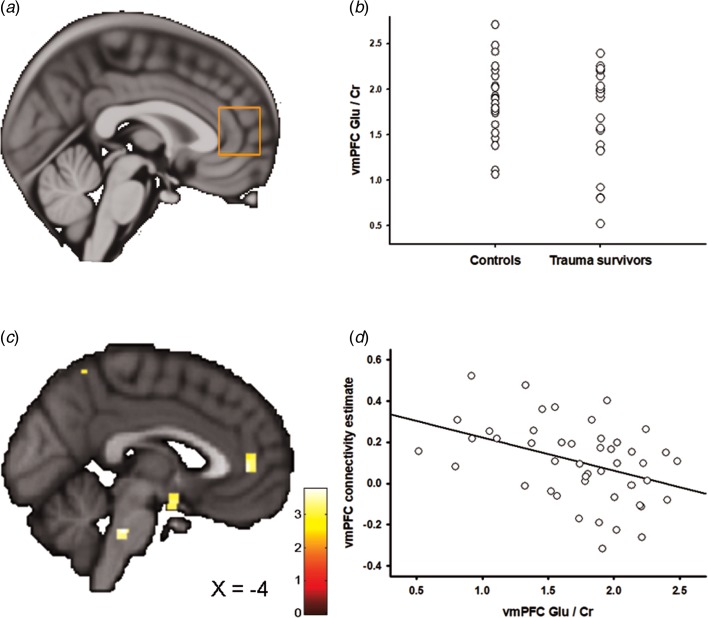
Ventromedial prefrontal cortex (vmPFC) glutamate and the association with amygdala–vmPFC connectivity. (*a*) Placement of the ^1^H-MRS voxel in vmPFC. (*b*) The group difference in vmPFC Glu/Cr. (*c*) Statistical parametric maps (SPM) demonstrating the cluster in vmPFC which was negatively associated with individual vmPFC Glu/Cr levels. The image is displayed at an uncorrected *p* = 0.005 and *k* = 25 for illustrative reasons. (*d*) Scatter plot illustrating the association between individual vmPFC Glu/Cr levels and amygdala–vmPFC connectivity. The vmPFC connectivity estimates were extracted from the group peak-activation voxel.

**Fig. 3.**
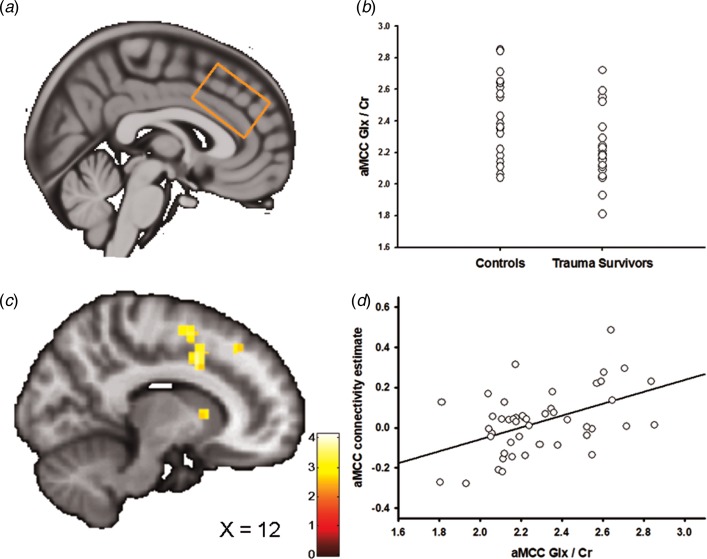
Anterior midcingulate cortex (aMCC) Glx and the association with amygdala–aMCC connectivity. (*a*) Placement of the ^1^H-MRS voxel in the aMCC cortex. (*b*) The group difference in aMCC Glx/Cr. (*c*) Statistical parametric maps (SPM) demonstrating the cluster in aMCC which was positively associated with individual aMCC Glx/Cr levels. The image is displayed at an uncorrected *p* = 0.005 and *k* = 25 for illustrative reasons. (*d*) Scatter plot illustrating the association between individual aMCC Glx/Cr levels and amygdala–aMCC connectivity. The aMCC connectivity estimates were extracted from the group peak-activation voxel.

Figures 2*a* and 3*a* show the positioning of the vmPFC ^1^H-MRS and the aMCC ^1^H-MRS voxels, respectively. We recently reported that the trauma survivors exhibited reduced levels of Glx/Cr in the aMCC, with no group differences revealed for vmPFC Glx/Cr (Ousdal *et al*., 2017). In the present study, we additionally compared Glu levels *per se* between the groups for the vmPFC and the aMCC voxel. Group was significantly associated with vmPFC Glu/Cr (two-way analysis of covariance controlling for voxel grey and white matter: *F*_(3,46)_ = 4.54, *p* = 0.04, partial *η*^2^ = 0.10, Fig. 2*b*). However, there was no significant group effect for Glu/Cr (*t*_45_ = 0.38, *p* = 0.71) in the aMCC. Mean Glu/Cr values for the vmPFC and the aMCC voxels are presented in online Supplementary Table S4.

In two separate *post*-*hoc* analyses, we explored whether the glutamatergic compounds which displayed significant group differences also predicted inter-individual differences in amygdala functional connectivity. In the first analysis, we regressed individual vmPFC Glu scores onto the individual functional connectivity maps while controlling for vmPFC white and grey matter. The analysis revealed a negative association between vmPFC Glu and amygdala–vmPFC functional connectivity (*x* = −6, *y* = 44, *z* = 4, *t* = 3.60, *p*_SVC_ = 0.02, Fig. 2*c*, *d*); however, this association only approached significance after controlling for group (*x* = −4, *y* = 44, *z* = 4, *t* = 3.16, *p*_SVC_ = 0.06), and should therefore be interpreted with caution. Based on a previous finding of reduced levels of aMCC Glx in the trauma survivors (Ousdal *et al*., 2017), a subsequent regression of individual aMCC Glx levels onto the functional connectivity maps revealed exactly one whole-brain significant cluster, which was localized in the aMCC (*x* = 12, *y* = 8, *z* = 38, *t* = 4.15, *p*_FWE_ = 0.02, Fig. 3*b*–*d*). The association remained nominal significant (*x* = 12, *y* = 8, *z* = 38, *t* = 3.48, *p*_SVC_ = 0.04) after controlling for the effect of group.

If the neurochemical changes were related to the traumatic event, we would expect an association between the individual metabolite levels and time elapsed since the trauma. Indeed, there was a negative association between vmPFC Glu levels and time since trauma (partial correlation controlling for vmPFC grey and white matter: *r* = −0.51, *p* = 0.02), supporting that the reduction of vmPFC Glu developed over time. Previous analyses revealed no association between aMCC Glx and time elapsed since the traumatic event (Ousdal *et al*., 2017).

### Combined analysis of fMRI–^1^H-MRS data

**Fig. 4.**
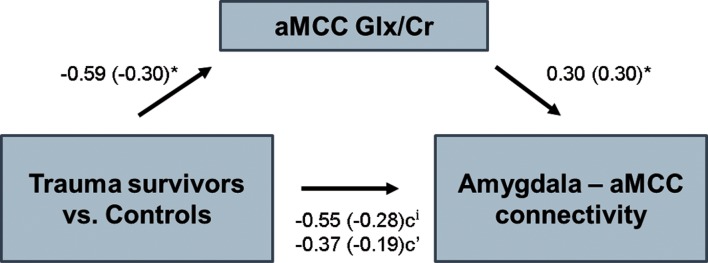
Mediation analysis. aMCC Glx/Cr levels mediated the relationship between group and amygdala–aMCC functional connectivity. *p < 0.05, ^i^*p* < 0.05, one-sided. Standardized coefficients in parenthesis.

We have previously reported that subjects in the trauma group had significantly lower levels of Glx in the dACC/aMCC (Ousdal *et al*., 2017). Moreover, we now report an association between individual levels of aMCC Glx and amygdala–aMCC connectivity. The findings suggest that traumatic stress exposure may affect amygdala–medial prefrontal connectivity through an impact on prefrontal glutamatergic metabolite levels. To further test this hypothesis, we examined whether aMCC Glx mediated the group difference in amygdala–aMCC functional connectivity. Using hierarchical regression, we first demonstrated that the group predicted amygdala–aMCC connectivity (*B* = −0.55, *t* = −1.92, *p* = 0.03, one-sided). A second regression showed that the group was associated with aMCC Glx (*B* = −0.59, *t* = −2.09, *p* = 0.04). aMCC Glx was also associated with amygdala–aMCC connectivity (*B* = 0.30, *t* = 2.03, *p* < 0.05). Importantly, adding the aMCC Glx levels as a second predictor of the amygdala–aMCC connectivity removed the effect of the group (*B* = −0.37, *t* = −1.30, *p* = 0.20), and the indirect effect of aMCC Glx on amygdala–aMCC connectivity was significant (bootstrap results for indirect effect; 95% CI [−0.50, −0.02], Fig. 4), consistent with a mediating role. For completeness of analyses, we also examined whether vmPFC Glu mediated the group difference in amygdala–vmPFC functional connectivity. The indirect effect of vmPFC Glu on amygdala–vmPFC connectivity was not significant (bootstrap results for indirect effect; 95% CI [−0.01, 0.06]), thus formal mediation was not supported.

## Discussion

We have shown that experiencing an episode of traumatic stress during late adolescence has long-term impact on amygdala–PFC neuronal circuitries. More specifically, survivors of the Norwegian terror attack showed less positive amygdala–aMCC connectivity and less negative amygdala–vmPFC connectivity compared with a matched group of young adults without any trauma exposure. Moreover, measurements of Glu in the vmPFC cortex indicated that trauma exposure led to long-term reduction in vmPFC Glu levels. This fits with the previous findings of reduced aMCC Glx levels in the trauma survivors (Ousdal *et al*., 2017). The finding of an association between vmPFC glutamatergic levels and time elapsed since the traumatic event supports that these changes in medial prefrontal neurochemistry were related to the trauma *per se*, and may evolve with increasing chronicity of the post-traumatic reactions (Popoli *et al*., 2012). Importantly, the group difference in amygdala–aMCC connectivity was mediated by aMCC Glx, suggesting that traumatic stress may shape the amygdala–medial PFC circuitry through an impact on prefrontal glutamate and its compounds.

The present results support previous findings of altered connectivity between prefrontal cortical regions implicated in emotional regulation (i.e. vmPFC) and the amygdala in the aftermath of significant stress exposure (Gee *et al*., 2013; Thomason *et al*., 2015). vmPFC–amygdala connections have found to be negative during rest (Jalbrzikowski *et al*., 2017) and during implicit emotional regulation (Etkin *et al*., 2006) in functional imaging studies, which is proposed to reflect top-down inhibitory regulation of amygdala activity (Etkin *et al*., 2011). Accordingly, loss of this top-down inhibition leads to strengthening of amygdala-dependent fearful behaviours in animal models (Adhikari *et al*., 2015). Similar behavioural changes can be observed in humans exposed to traumatic stressors (Tottenham *et al*., 2010), and in patients with stress-related mental illness (Milad *et al*., 2009) alongside exaggerated amygdala and dampened vmPFC activity (Milad *et al*., 2009; Pitman *et al*., 2012). Thus, we speculate that adverse exposures impacting on these top-down inhibitory pathways during adolescence increases vulnerability for stress-related mental illness through an effect on emotion regulation abilities.

In addition to reduced negative connectivity between vmPFC and the amygdala, we found significantly reduced positive connectivity between the amygdala and aMCC in the trauma survivors. The result is in line with studies in subclinical and clinical PTSD groups also finding compromised resting-state amygdala–dACC/aMCC connectivity (Thomason *et al*., 2015; Wolf and Herringa, 2016), although the level of disconnection may depend on the individual depressive symptom load (Satterthwaite *et al*., 2016). Whereas vmPFC plays a key role in emotional regulation, dACC/aMCC is preferentially involved in the expression and acquisition of emotions (Etkin *et al*., 2011), and thus show positive functional connectivity with the amygdala during rest (Kerestes *et al*., 2017). Although mostly linked to emotion expression, these connections may exert inhibitory control over amygdala activity in situations requiring explicit emotional regulation (Etkin *et al*., 2011; Gyurak *et al*., 2011), which is likely to occur indirectly through the vmPFC. In line with this model, and previous studies of trauma-exposed youths (Thomason *et al*., 2015), our results show consistent between-group effects with regards to the amygdala–vmPFC and the amygdala–aMCC connectivity. Moreover, the notion that trauma had distinct modulatory effects on the various amygdala–prefrontal neurocircuits could help explain why trauma affects aspects of both fear learning and fear extinction (Maren and Holmes, 2016). Interestingly, both the amygdala and the cingulate cortex influence autonomic arousal (Luu and Posner, 2003). Although speculative, the autonomic hyper-responsiveness in traumatized individuals may thus be linked to altered amygdala–cingulate connectivity following trauma (Thomason *et al*., 2015).

Mounting evidence from animal studies suggests that acute and chronic stress affect Glu neurotransmission in the PFC (Popoli *et al*., 2012). While acute stress has been shown to enhance Glu and prefrontal cognitive functions, repeated exposure to stress, as well as long-term effects of some acute stressors bring about structural changes and diminished Glu neurotransmission in animal models (Popoli *et al*., 2012; Yuan and Hou, 2015). A few studies have reported reduced dACC/aMCC Glu in patients with established PTSD following trauma exposure (Pennington *et al*., 2014; Yang *et al*., 2015), although, to the best of our knowledge, no study has investigated vmPFC Glu levels in relation to a stressful experience. While we found reduced levels of Glu in vmPFC, the group effect was revealed in a summary measure of Glu and its metabolic compound, glutamine, in the aMCC (Ousdal *et al*., 2017). This anatomical discrepancy is possible, given the regional difference within the cingulate cortex receptor densities and hence glutamate turnover (Palomero-Gallagher *et al*., 2009; Dou *et al*., 2013), but could also reflect a subtle association between trauma and glutamatergic neurotransmission. Indeed, traumatic stress is likely to affect numerous neurotransmitters (Pitman *et al*., 2012), and thus the effects of trauma on glutamatergic metabolite concentrations may be secondary to more robust effects on these other neurochemicals. Furthermore, the inherently limited spatial resolution of ^1^H-MRS, which restricts measurements to ‘bulk’ levels of metabolites, and the technical challenges especially related to acquisitions in the most ventral parts of the PFC (de Matos *et al*., 2016), may also explain the regional differences in metabolite levels. Irrespective of this, the present results support previous findings of reduced prefrontal glutamatergic metabolite levels following traumatic stress (Pennington *et al*., 2014; Yang *et al*., 2015), and also expand these findings by linking individual prefrontal glutamatergic metabolite levels to long-range amygdala–prefrontal functional connectivity.

The directionality of the associations is worth reiterating. We have previously reported that traumatic stress was associated with a reduction of aMCC Glx (Ousdal *et al*., 2017). As aMCC Glx levels were positively associated with amygdala–aMCC connectivity, this not only correctly predicted the reduced amygdala–aMCC connectivity in trauma survivors, but a mediation analysis also provided direct evidence for the involvement of aMCC Glx levels in mediating the impact of trauma on connectivity. In addition to the statistical coherence, these results are also biologically plausible as chronic or traumatic stress is associated with PFC architectural changes (Arnsten, 2015; McEwen *et al*., 2015), which could directly impact long-range glutamatergic connections. Although speculative, a reduction in aMCC glutamate transmission may decrease the synaptic strength of aMCC efferents onto amygdala neurons reflected in a loss of positive amygdala–aMCC connectivity.

We acknowledge a potential limitation of the present study rests in the relative small group sizes and the heterogeneity related to the trauma group. This is always likely to be a problem in these types of studies given the variability of response to stressors. Furthermore, we acknowledge that the use of an amygdala ROI and not an independent component analysis when processing the rsfMRI data may be less sensitive to physiological noise (Van Dijk *et al*., 2010). To address this, we applied rigorous methods to detect and remove physiological noise and movement-related artefacts during preprocessing and subsequent data analysis. However, the use of a seed-based approach is likely to provide a more precise and detailed estimate of amygdala connectivity, which was the goal of the present study (Margulies *et al*., 2007). Moreover, accurate discrimination between Glu and glutamine can be difficult at 3 T, although data quality and fit reliability estimates indicated that the Glu measurement could be meaningfully reported in this case. The group difference in amygdala–vmPFC connectivity was not mediated by the group difference in vmPFC Glu levels. While it is possible that perturbations of amygdala–vmPFC connectivity following trauma is not driven by glutamatergic mechanisms, technical issues related to MRS acquisition combined with a modest sample size may have precluded finding significant mediation. Alternatively, disturbances in long-range amygdala–vmPFC glutamatergic connections may be related to the development of stress-related psychopathology (Horn *et al*., 2010) and not a consequence of stress exposure *per se*. As such, future studies should investigate potential associations between vmPFC Glu and amygdala connectivity in both PTSD patients and trauma-exposed control groups, to understand the association between stress exposure, stress-related psychopathology and amygdala–vmPFC glutamatergic connections. Finally, the association between amygdala functional connectivity and prefrontal glutamatergic metabolites were based on correlations, which do not imply causation. Thus, the present results should be interpreted with caution, bearing in mind the study design and analytic approaches.

In conclusion, we found that traumatic stress influences functional connectivity between amygdala and medial prefrontal cortical regions, which are regions implicated in emotion generation and regulation. More specifically, trauma-exposed individuals had less positive amygdala–aMCC connectivity and less negative amygdala–vmPFC connectivity compared with a matched group without any trauma exposure. Overall, the results support a model in which traumatic stress is associated with reduced regulation of amygdala responses, both directly and indirectly. The amygdala–aMCC connectivity pattern was mediated by Glx, suggesting that the compromised connectivity in trauma survivors may be secondary to trauma-induced changes in prefrontal glutamatergic pathways. Identifying the neurochemical underpinning of the observed connectivity changes in trauma-exposed individuals may ultimately contribute to new pharmacological treatments of stress-related mental illness.

## Supplementary material

For supplementary material accompanying this paper visit https://doi.org/10.1017/S0033291718002519. click here to view supplementary material
