# Supplementary material for: Prefrontal glutamate levels predict altered amygdala–prefrontal connectivity in traumatized youths
Source: Psychol Med. 2018 Sep 18;49(11):1822–30. doi: 10.1017/S0033291718002519 (PMC6650776; doi:10.1017/S0033291718002519)
Supplement: Supplementary file 1 [file S0033291718002519sup001.docx]

# Prefrontal glutamate levels predict altered amygdala – prefrontal connectivity in traumatized youths

# Supplemental Information

## Supplementary Method

### MR image acquisition parameters

Whole-brain, T2*-weighted, echo-planar (EPI) images (TR= 2000 ms, TE= 25 ms, Flip angle 90º, 260 mm x 260 mm field of view, 64 x 64 matrix) were acquired using a GE Signa HDx 3T MR scanner (General Electric Company; Milwaukee, WI, USA) supplied with a standard eight-channel head coil. A total of 160 volumes were acquired in addition to 5 dummy volumes which were discarded, to allow for magnetization equilibrium. Each volume consisted of 36 slices acquired parallel to the AC-PC plane (interleaved acquisition; 3.5 mm thick with a 0.5 mm gap). Participants were instructed to relax with their eyes closed.

For anatomical comparison purposes, FSPGR T1-weighted anatomical images (TR = 7.8 ms, TE = 3.0 ms, Flip angle 14º, 256 mm x 256 mm field of view, 256 x 256 matrix) were acquired prior to the functional imaging.

### Magnetic resonance spectroscopy (^1^H-MRS) acquisition and analysis.

^1^H-spectra were obtained from the ventromedial prefrontal cortex (vmPFC) and dorsal anterior cingulate cortex (dACC)/ anterior midcingulate cortex (aMCC) using a single voxel point resolved spectroscopy (PRESS) sequence (TR = 1500 ms, TE = 35 ms, 128 averages). The two MRS voxels were positioned from a high-resolution T1-image, across the midline, covering the vmPFC (voxel size 25 x 40 x 40 mm) and the aMCC (voxel size 30 x 40 x 25 mm) respectively. We used a standard 15-component basis set. The quality of the spectra was ensured by applying pre-scanning iteratively to achieve water suppression level at minimum 94% and a line-width of less than 8 Hz. The Linear Combination Modelling (LCModel) software (Version 6.2-4A (Provencher 1993)) was used for quantification of the ^1^H-MRS spectra. We excluded spectra with a Cramer-Rao minimum variance bounds (CRLB) >20 or with aberrations in the spectrum of residuals, determined on visual inspection guided by local quality control algorithms.

Resting-state Glu and Glx levels relative to Creatine (Cr) were used from the LCModel output. Cr levels in aMCC (t_(45)_ = 0.58, p=0.56) or vmPFC (t_(45)_ = -0.45, p=0.66) did not differ significantly between the two groups. Moreover, there was no difference in the quality of LC model fits for the two groups as indexed by the signal-to-noise ratio for the aMCC (t_(45)_ = -0.34, p = 0.74) or the vmPFC (t_(45)_ =-0.26, p = 0.80) voxel.

As glutamate levels might depend on the amount of grey or white matter within the voxel, we additionally tested whether anatomical differences within the voxels impacted on our results. The proportion of grey and white matter in each voxel was calculated using in-house written Matlab scripts that leverage the SPM8 software’s (http://www.fil.ion.ucl.ac.uk/spm) combined segmentation and normalization functions. Coordinates and orientation in scanner space was read directly from the raw spectroscopy files. Subsequently, the T1 image was co-registered to a standard MNI template and segmented into tissue probability maps for three classes of tissue (grey matter, white matter and CSF). Ventromedial PFC (vmPFC) Glu/Cr levels were associated with voxel grey (r=0.33, p = 0.02) and white (r=-0.31, p = 0.03) matter. Accordingly, we corrected for voxel grey and voxel white matter in all analyses of these data. Similar analyses for vmPFC Glx/Cr (grey matter: r=-0.28, p = 0.06, white matter: r=-0.25, p = 0.09), aMCC Glu/Cr (grey matter: r=-0.20, p = 0.17, white matter: r=0.24, p = 0.10) or aMCC Glx/Cr (grey matter: r=0.17, p = 0.26, white matter: r=0.12, p = 0.41) levels did not reach significance.

## Supplementary Table 1

The table shows the results from a one-sample t-test contrasting amygdala functional connectivity maps across all participants.

| Region | L/R | *x* | *y* | *z* | *T* | *P^a^* |
| --- | --- | --- | --- | --- | --- | --- |
| Medial temporal lobe | R | 24 | -2 | -20 | 48.34 | <0.001 |
| Medial temporal lobe | L | -24 | -2 | -20 | 44.83 | <0.001 |
| Anterior midcingulate cortex / dorsal anterior cingulate cortex | R | 10 | -4 | 50 | 5.17 | <0.001 |
| Ventromedial prefrontal cortex | R | 6 | 50 | -22 | 4.88 | <0.001 |
| Medial frontal gyrus | R | 4 | 52 | 28 | 4.80 | 0.04 |

^a^FWE cluster level significance

## Supplementary Table 2

The table shows the results from a multiple regression analysis assessing the association between groups (i.e trauma survivors vs. controls) and amygdala functional connectivity while controlling for comorbid post-traumatic stress disorder (PTSD) and panic disorder.

| Region | L/R | *x* | *y* | *z* | *T* | *P^a^* |
| --- | --- | --- | --- | --- | --- | --- |
| Anterior midcingulate cortex / Supplementary motor cortex | R | 6 | -10 | 62 | 5.99 | <0.001 |
| Ventromedial prefrontal cortex | L | -18 | 40 | -10 | 5.20 | <0.001 |
| Dorsolateral prefrontal cortex | R | 40 | -20 | 38 | 4.29 | 0.001 |
| Cerebellum | L | -26 | -58 | -62 | 4.10 | 0.04 |

^a^FWE cluster level significance

## Supplementary Table 3

The table shows the results from a two-sample t-test comparing amygdala functional connectivity between trauma survivors and controls in un-scrubbed (i.e. without interpolation) data.

| Region | L/R | *x* | *y* | *z* | *T* | *P^a^* |
| --- | --- | --- | --- | --- | --- | --- |
| Anterior midcingulate cortex | R | 6 | -10 | 62 | 5.99 | <0.001 |
| Cerebellum | L | -4 | -56 | -20 | 4.29 | <0.001 |
| Ventromedial prefrontal cortex | L | -14 | 38 | 10 | 4.58 | 0.004 |
| Supramarginal gyrus | L | -62 | -35 | 28 | 4.05 | 0.008 |

^a^FWE cluster level significance

## Supplementary Table 4

^1^H-MRS-values from the voxel in aMCC and vmPFC divided by group.

|  | aMCC | | vmPFC | |
| --- | --- | --- | --- | --- |
|  | Trauma | Controls | Trauma | Controls |
| Glu/Cr | 1.85 ± 0.17 | 1.87 ± 0.14 | 1.63 ± 0.56 | 1.86 ± 0.39 |
| GM | 42.95 ± 3.06 | 43.43 ± 4.23 | 37.16 ± 6.18 | 38.30 ± 5.12 |
| WM | 48.86 ± 3.44 | 47.98 ± 3.90 | 50.94 ± 4.46 | 49.92 ± 3.98 |

± indicates SD

Abbreviations: Glu/Cr = resting-state Glutamate relative to Creatine. GM = Grey matter proportion in voxel; WM = White matter proportion in voxel.

## Supplementary Figure 1

Statistical Parametric Map (SPM) demonstrating amygdala functional connectivity across groups. Images are whole-brain FWE cluster corrected. See also Supplementary Table 1.

#
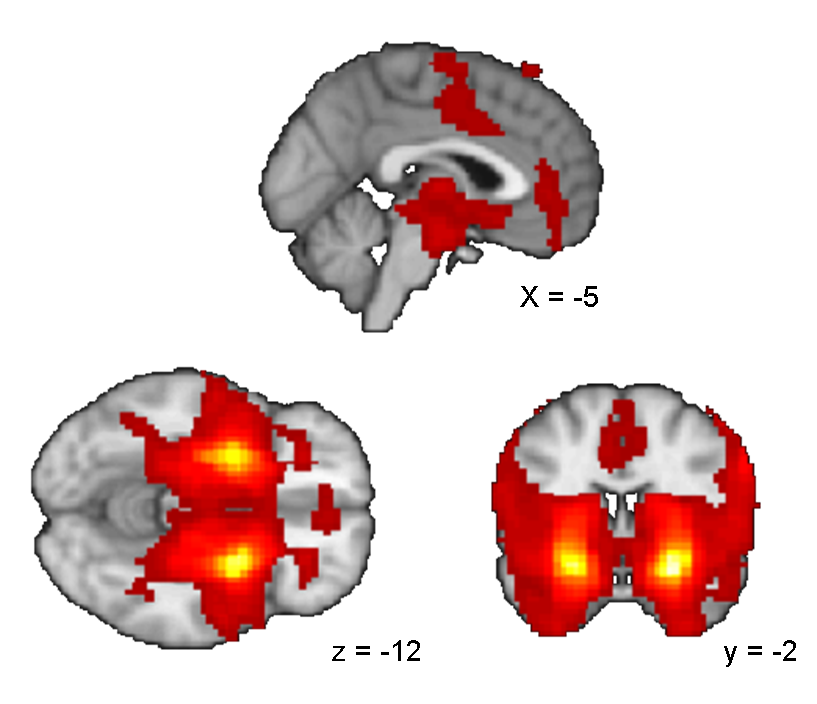


## Supplementary Figure 2

Overlap between the aMCC and the vmPFC clusters identified from the group comparison of amygdala functional connectivity and the medial prefrontal MRS voxels. A: Overlap between the aMCC MRS voxel and the aMCC cluster. The SPM image (violet) is displayed at an uncorrected p<0.005 for illustrative reasons. The aMCC MRS voxel is displayed in green-blue colour indicating the probability distribution for the anatomical placement across subjects. Green indicates regions covered by at least 50% of placements, while blue indicates regions covered by at least 95% of placements. B: Overlap between the vmPFC MRS voxel and the vmPFC cluster. The SPM image (violet) is displayed at an uncorrected p<0.005 for illustrative reasons. The vmPFC MRS voxel is displayed in green-blue colour indicating the probability distribution for the anatomical placement across subjects. Green indicates > 50% coverage and blue > 95% coverage.


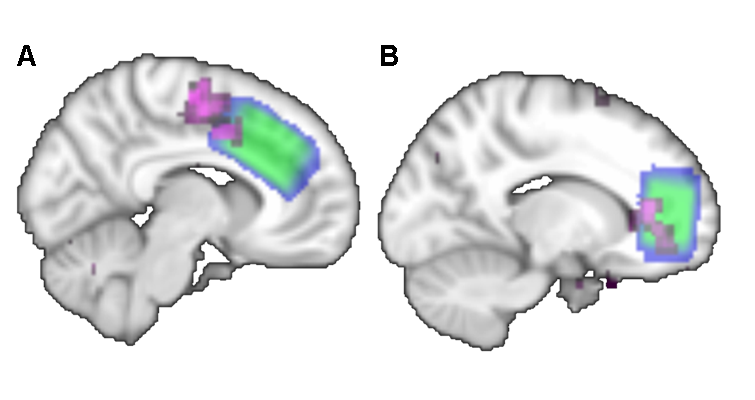


# REFERENCES

**Provencher SW** (1993). Estimation of metabolite concentrations from localized in vivo proton NMR spectra. *Magnetic Resonance Medicine* **30**, 672-679.
